# Supplementary material for: Tgfbr1 controls developmental plasticity between the hindlimb and external genitalia by remodeling their regulatory landscape
Source: Nat Commun. 2024 Mar 20;15:2509. doi: 10.1038/s41467-024-46870-z (PMC10954616; doi:10.1038/s41467-024-46870-z)
Supplement: Supplementary file 1 — Supplementary Information [file 41467_2024_46870_MOESM1_ESM.pdf]

Supplementary information

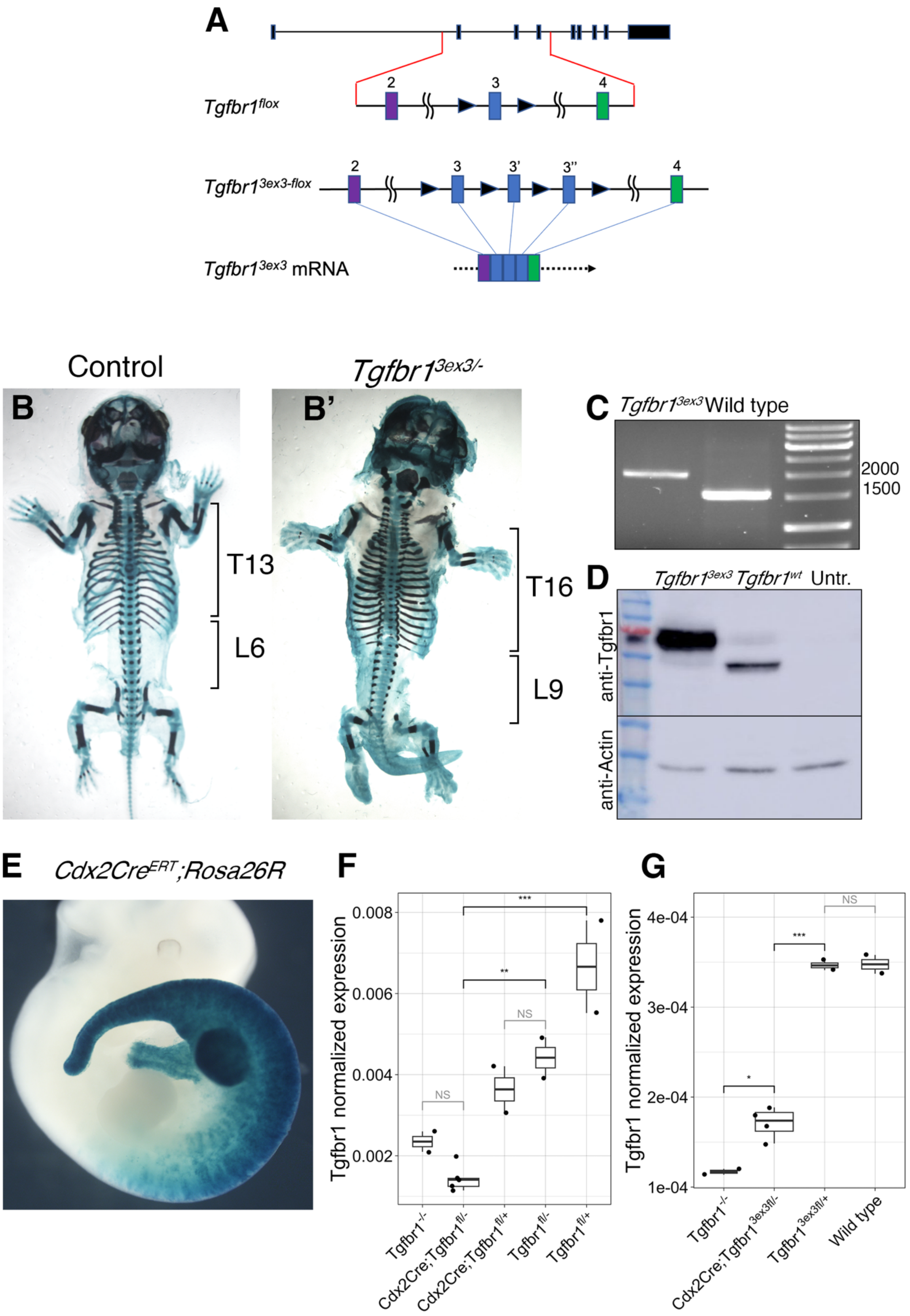

Supplementary Fig. 1. Generation on the *Tgfbr1-cKO*. (A) Scheme showing *Tgfbr1*^{flox} and *Tgfbr1*$^{3ex3\text{-}flox}$ alleles. Highlighted is the region containing exons 2, 3 and 4 (colored boxes) and the LoxP sites (black triangles). Also represented is the generation of a mRNA with a triplicated exon 3 after splicing of the transcript from the *Tgfbr1*$^{3ex3\text{-}flox}$ allele. (B, B') Skeletal staining of a E17.5 wild type (B) and *Tgfbr1*$^{3ex3\text{-}flox/-}$ fetus (B'), showing the presence of additional thoracic (T) and lumbar (L) vertebrae, resembling a *Gdf11* mutant phenotype. (C) RT-PCR fragment spanning the whole *Tgfbr1* coding region using RNA isolated from wild type (1,5 kb) and *Tgfbr1*$^{3ex3\text{-}flox/3ex3\text{-}flox}$ (2 kb) embryos. (D). Western blot analysis of protein extracts from HEK293T cells transfected with mammalian expression vectors carrying either the *Tgfbr1*^{3ex3} or the *Tgfbr1* coding regions, showing that the *Tgfbr1*^{3ex3} transcript produces a larger protein than its wild type counterpart. (E) Activation of β-galactosidase expression in a tamoxifen-treated E10.5 *Cdx2Cre*^{ERT}*;Rosa26R* embryo following the scheme used in this study. (F) RT-qPCR showing recombination efficiency in *Tgfbr1*$^{flox/-}$*;Cdx2Cre*$^{ERT+/0}$ embryos. Normalized *Tgfbr1* expression level in *Tgfbr1*$^{flox/-}$ *::Cdx2Cre*$^{ERT+/0}$ is not different from *Tgfbr1*$^{-/-}$. Level of normalized expression in *Tgfbr1*$^{+/flox}$*;Cdx2Cre*$^{ERT+/0}$ equals that of *Tgfbr1*$^{flox/-}$ and half of *Tgfbr1*$^{flox/+}$ control, consistent with it carrying only one functional allele (n=2 control and n=5 *Tgfbr1-cKO* E10.5 embryos were used per genotype. P-values were determined using Tukey's post hoc tests after one-way ANOVA). **p= 0.008; mean=2.971e-3, 95%CI [8.550e-4, 5.088e-3], ***p< 0.001, mean=5.214e-3, 95%CI [3.098e-3, 7.33e-3]. (G) RT-qPCR showing recombination efficiency in *Tgfbr1*$^{3ex3\text{-}flox/-}$*;Cdx2Cre*$^{ERT+/0}$ embryos. Normalized *Tgfbr1* expression level in *Tgfbr1*$^{3ex3\text{-}flox/-}$*;Cdx2Cre*$^{ERT+/0}$ embryos is significantly lower than in control embryos, although not reaching *Tgfbr1*$^{-/-}$ level, consistent with lower amount of tamoxifen given compared to the *Tgfbr1*^{flox} line. (n=2 control and n=4 *Tgfbr1-cKO* E10.5 embryos were used per genotype. P-values were determined using Tukey's post hoc tests after one-way ANOVA). *p= 0.018; mean= 5.416e-05, 95%CI [1.129e-05, 9.703e-05], ***p <0,001; mean= 1.752e-04, 95%CI [1.323e-04, 2.181e-04]. NS: nonsignificant. Source data are provided as a Source Data file.

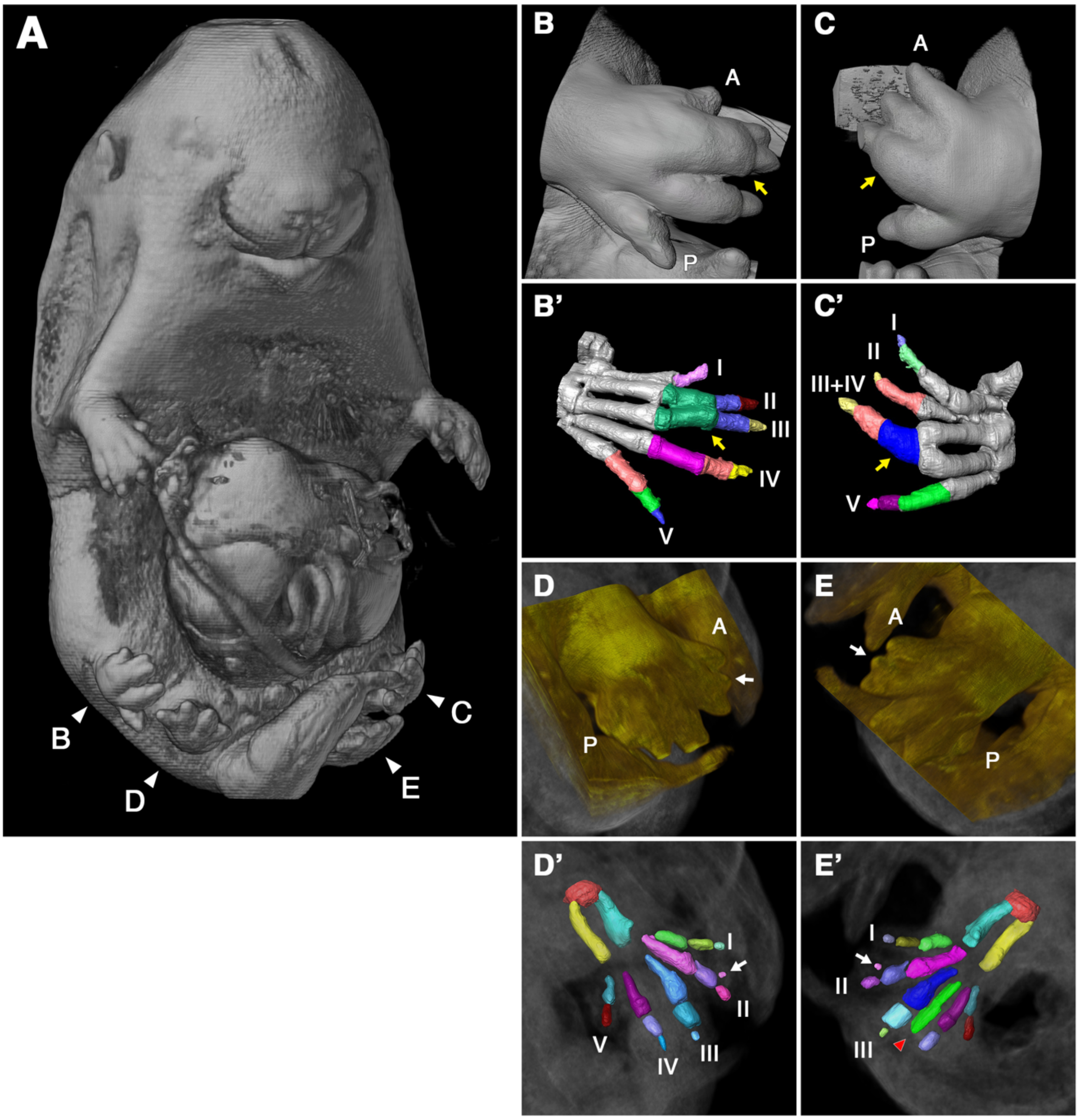

Supplementary Fig. 2. Skeletal phenotype of the *Tgfbr1-cKO* native (B-C') extra (D-E') hindlimbs. (A) Whole mount image of the OPT acquired E16.5 fetus. (B, B') Whole mount (B) and 3D reconstruction of the skeleton (B') of the right hindlimb. The autopod formed 5 digits. Digit I had 1 distal phalanx, and digits II to V three phalanxes each. Syndactyly between digits II and III (yellow arrow) was also observed. (C, C') Whole mount (C) and 3D reconstruction of the skeleton (C') of the left hindlimb. The autopod formed 5 malformed digits. Digits I and II contained two phalanxes each. The other digits had three phalanxes. The phalanges of digits III and IV were fused (yellow arrow). (D, D') Whole mount (D) and 3D reconstruction of the skeleton (D') of the right extra hindlimb. The autopod contained 5 digits, most of them with two phalanges, except for digit V, which only generated one phalanx and digit II, which contained two adjacent distal phalanges (white arrow in D and D'). (E, E') Whole mount (E) and 3D reconstruction of the skeleton (E') of the left extra hindlimb. The autopod contained 6 metatarsal bones. Anterior digits developed two phalanges, except for digit II, which generated two adjacent distal phalanges (white arrow in E and E'); the posterior digits developed one phalanx, except for one digit that did not develop any distal phalanges (red arrowhead in E'). A – anterior, P – posterior.

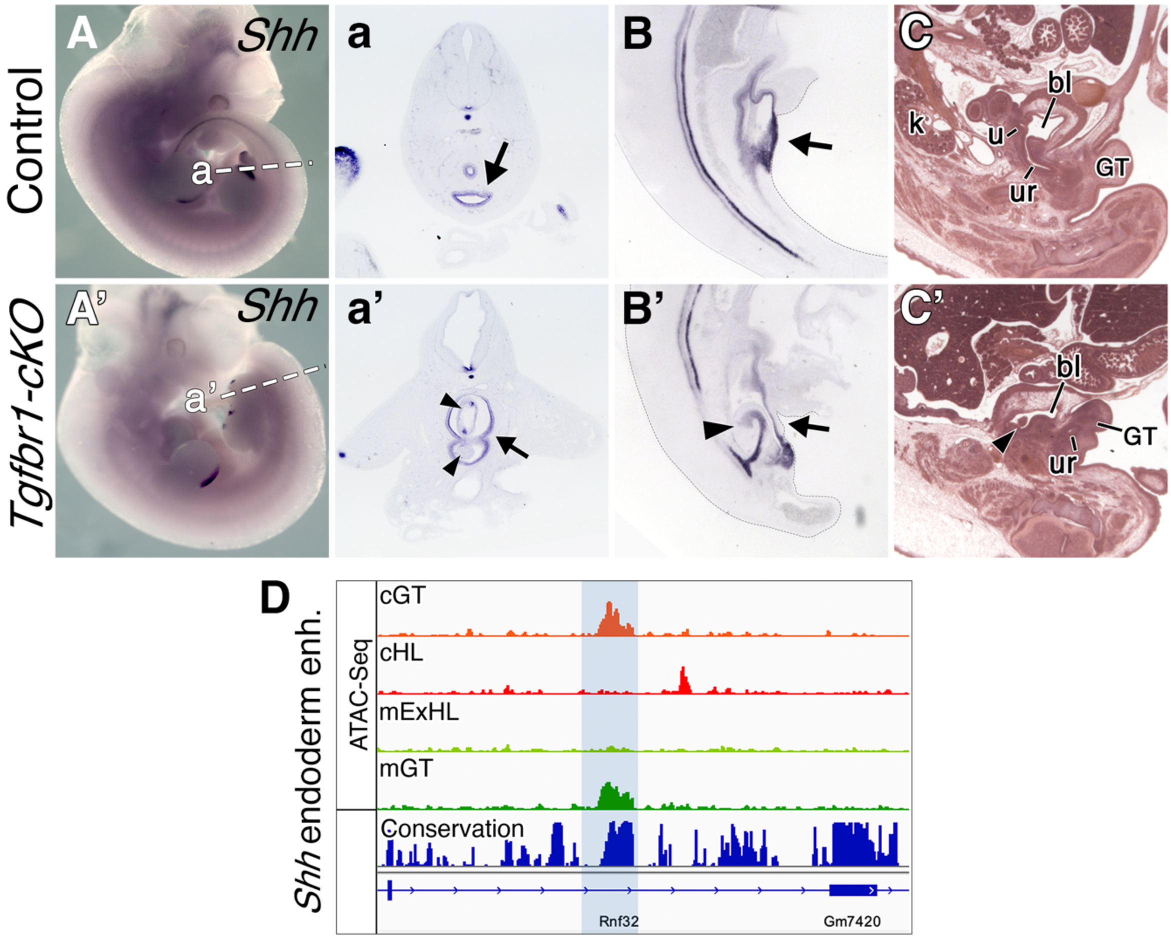

Supplementary Fig. 3. Urogenital malformations in the *Tgfbr1-cKO.* (A-a') *Shh* expression in E11.5 control (A, a) and *Tgfbr1-cKO* (A', a') embryos. (a, a') show transversal sections through the cloacal region at the level indicated by the dashed line in A and A'. (B, B') Sagittal sections through E10.5 control (B) and *Tgfbr1-cKO* (B') embryos stained with a *Shh* probe. (C, C') Sagittal histological sections through bladder region of E16.5 control (C) and *Tgfbr1-cKO* (C') fetuses. Black arrows in a, a', B, B' indicate the cloaca; arrowheads in a', B', C' indicate intra-cloacal protrusions; bl: bladder; GT: genital tubercle; k: kidney; u: ureter; ur: urethra. (D) ATAC-Seq profiles through the region containing the enhancer regulating endodermal *Shh* expression (blue shadow). Bottom track shows conservation in placental mammals. cHL: control hindlimb; mExHL: mutant extra hindlimb; cGT: control GT; mGT: mutant GT.

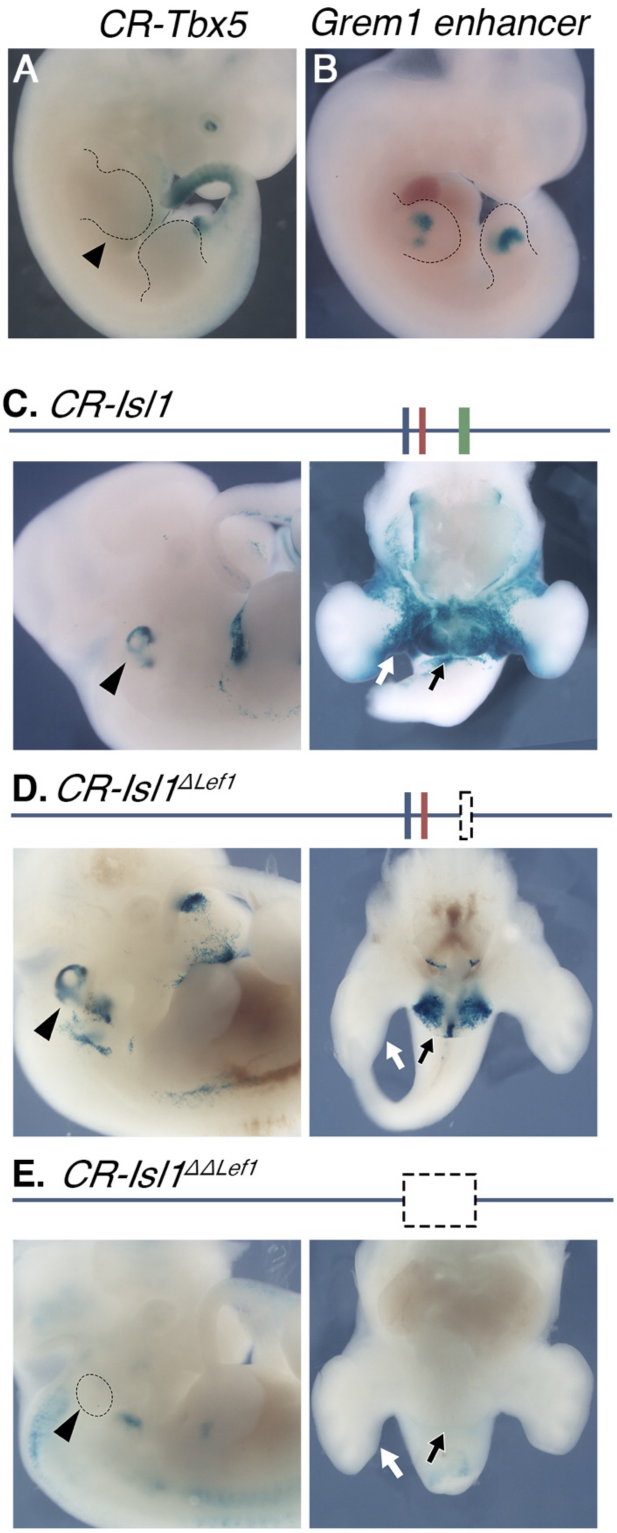

Supplementary Fig. 4. Characterization of *CR-Isl1* (A) The putative *Tbx5* GT enhancer shown in Fig. 4I' does not drive β-galactosidase expression in the forelimb (black arrowhead, forelimb outlined with the dashed line). (B) β-galactosidase expression driven by the *Grem1* enhancer in Fig. 5D using a transgenic reporter assay [n=2/8] (limbs outlined with the dashed line). (C-E) Activation of the β-galactosidase reporter by the *CR-Isl1* (C), *CR-Isl1*$^{\Delta Lef1}$ (D), and *CR-Isl1*$^{\Delta\Delta Lef1}$ (E) region in E12.0 embryos. Of note, the embryo shown in D is the one with the strongest reporter expression in the GT region of all *Isl1*$^{\Delta Lef1}$

transgenic embryos analyzed. Top panels show schematic representation of DNA region: blue rectangle: 5' Lef1 binding motif; red rectangle: Gata3 binding motif; green rectangle: 3' Lef1 binding motif within the TF footprint observed in the *Isl1*-related Pattern 1 element. Black arrowheads show expression in otic vesicle; white arrow shows expression in posterior limb bud; black arrow indicates expression in GT. CR: conserved region.

Supplementary Table 1. Oligonucleotides and probes used in this work.

**Genotyping primers**		
Tgfbr1-flox	Forward	TTGAGCTTGCTGTCTGACTGGATAG
	Reverse	CTGTGGTTGGCAGGCATGTG
Cre	Forward	CGAGTGATGAGGTTCGCAAG
	Reverse	CCTGATCCTGGCAATTTCGGCT
Tgfbr1-null	Forward	CTACTGTGTTTCAAATGGGAGGGC
	Reverse	GGCCTGTCGGATCCTATCATC
β-galactosidase	Forward	AGCAGTTTTTCCAGTTCCGTTTATC
	Reverse	AGCGGCGTCAGCAGTTGTTTTTTAT
**Cloning of regulatory elements**		
CR-Isl1	Forward	GACTCGAGTCTGTGATACAAAACAATATATC
	Reverse	GAGGATCCAATCTTTCAAAGACATGGAGGG
Isl1 - Left1 sites deletions	Primer1	GCGAATTCGGCTGAATGGAGGTCATTGGA
	Primer2	GCGAATTCTTCTGGCTTCAGTTCCTGAGAC
	Primer3	GCGAATTCCAGGTATGAAAAGCTGTGC
CR-Tbx5	Forward	GACTCGAGCTTTATGTATCTGAGCACACTG
	Reverse	GAAGATCTTCCTTCAACAAACCAT CCAC
CR-Wnt5a	Forward	CTGCTCGAGACTAGTACCTGCTGCTTATCAACCTAGTG
	Reverse	CAGCCATGGCTCCTACCTCACTGACTATCCAG
CR-Fgf10	Forward	CAGCTCGAGACTAGTAATCTGAGGTGGTTGCTCACTC
	Reverse	CATCCATGGCTATGGAAGGCTTATGTATCAC
Grem1-Enh	Forward	GACGTCGACACTAGTGTCATGTGTTCCTGTGATTCTG
	Reverse	GAGCCTGTATAAGAAGTTCAGGGCCA TGGCAG
**Other primers**		
qPCR-Tgfbr1	Forward	ACCGTGTGCCAAATGAAGAGG
	Reverse	CATCTAGATCTTGTAACACAATGGTCCTGGC
qPCR-Actin	Forward	ATGAAGATCCTGACCGAGCG
	Reverse	TACTTGCGCTCAGGAGGAGC
Tgfbr1-CDS	Forward	GAGAATTCCGGGCCACAAACAGTGGC
	Reverse	GAGTCGACCATTTTGATGCCTTCCTGT TGGC
**Oligonucleotides for generating in situ probes**		
Lmx1b	Forward	CTGCTGTGCAAGGGTGACTATGAG
	Reverse	GAGGCAAAGTAGGAGCTCTGCATG
Fgf10	Forward	TTCCTAGAAGTTATGGATGTTG
	Reverse	GTACTGCATCCACCAACAGTG
Grem1	Forward	AATGAATCGCACCGCATACAC

	Reverse	GACTAATACGACTCACTATAGGGAAGCAACTGCTGGTTCTTCTG
**In situ probes**		
En1	Davis and Joyner, *Genes Dev* **2**, 1736–1744 (1988)	
Fgf10	Whole coding region cloned into TOPO. Oligonucleotides listed above	
Fgf8	Crossley and Martin, *Development* **121**, 439–451 (1995)	
Grem1	PCR fragment with the whole coding region and T7 promoter at the 3' end. Oligonucleotides listed above	
Hand2	Srivastava et al, 1997, *Nat Genet* **16**, 154–160 (1997)	
Isl1	Jurberg et al. *Dev Cell* **25**, 451–462 (2013)	
Lin28a	Aires et al., *Dev Cell* **48**, 383–395 (2019)	
Lmx1b	PCR fragment encompassing nucleotides 504 to 1202 of the mRNA cloned into pKS bluescript. Oligonucleotides listed above.	
Pitx1	Szeto et al, *Genes Dev* **13**, 484–494 (1999)	
Shh	Echelard et al, *Cell* **75**, 1417–1430 (1993)	
Tbx5	Chapman et al, *Developmental Dynamics* **206**, 379–390 (1996)	
Wnt5a	Yamaguchi et al, *Development* **126**, 1211–1223 (1999)	

Supplementary Table 2. Coordinates of the genomic elements used in the reporter assays (from the GRCm38/mm10 reference genome).

Element	Coordinates
CR-Isl1	chr13:116,284,356-116,285,685
CR-Tbx5	chr5:119,701,323-119,702,815
CR-Wnt5a	chr14:28,412,522-28,413,803
CR-Fgf10	chr13:118,745,999-118,747,518
Grem1 enhancer	chr2: 113,580,982-113,582,008
