# Supplementary material for: Tgfbr1 controls developmental plasticity between the hindlimb and external genitalia by remodeling their regulatory landscape
Source: Nat Commun. 2024 Mar 20;15:2509. doi: 10.1038/s41467-024-46870-z (PMC10954616; doi:10.1038/s41467-024-46870-z)
Supplement: Supplementary file 3 — Description of Additional Supplementary Files [file 41467_2024_46870_MOESM3_ESM.pdf]

Description of Additional Supplementary Files

File Name: Supplementary Data 1
Description: Transcription factor footprint analysis of pattern 1 elements. Column A: number of pattern 1 regions with at least 1 occurrence of the specified transcription factor; column B: number of pattern1 regions with no occurrence of the specified transcription factor; C: number of background regions with at least 1 occurrence of the specified transcription factor; D: number of background regions with no occurrence of the specified transcription factor. These data were generated by the program RGT HINT-ATAC without further adjustments, utilizing a statistical framework based on hidden Markov models (HMMs) (ref. 77).

File Name: Supplementary Movie 1
Description: OPT image from a E16.5 *Tgfbr1-cKO* fetus highlighting the limbs. The extra hindlimbs are shown in magenta.

File Name: Supplementary Movie 2
Description: OPT image from a E16.5 wild type fetus highlighting the caudal end of the intestine (red), the kidneys (cold green), the ovaries and uterus (bright green), the vagina (yellow), the urinary bladder (dark blue) and the urethra (light blue).

File Name: Supplementary Movie 3
Description: OPT image from a E16.5 *Tgfbr1-cKO* fetus highlighting the caudal end of the intestine (red), the urinary bladder (dark blue) and the urethra (light blue).
