# Supplementary material for: Tgfbr1 controls developmental plasticity between the hindlimb and external genitalia by remodeling their regulatory landscape
Source: Nat Commun. 2024 Mar 20;15:2509. doi: 10.1038/s41467-024-46870-z (PMC10954616; doi:10.1038/s41467-024-46870-z)
Supplement: Supplementary file 8 — Reporting Summary [file 41467_2024_46870_MOESM8_ESM.pdf]

nature portfolio

Corresponding author(s): Moises Mallo

Last updated by author(s): 03/03/2024

Reporting Summary

Nature Portfolio wishes to improve the reproducibility of the work that we publish. This form provides structure for consistency and transparency in reporting. For further information on Nature Portfolio policies, see our Editorial Policies and the Editorial Policy Checklist.

Statistics

For all statistical analyses, confirm that the following items are present in the figure legend, table legend, main text, or Methods section.

n/a	Confirmed	
	x	The exact sample size (*n*) for each experimental group/condition, given as a discrete number and unit of measurement
x		A statement on whether measurements were taken from distinct samples or whether the same sample was measured repeatedly
	x	The statistical test(s) used AND whether they are one- or two-sided<br>*Only common tests should be described solely by name; describe more complex techniques in the Methods section.*
x		A description of all covariates tested
x		A description of any assumptions or corrections, such as tests of normality and adjustment for multiple comparisons
	x	A full description of the statistical parameters including central tendency (e.g. means) or other basic estimates (e.g. regression coefficient) AND variation (e.g. standard deviation) or associated estimates of uncertainty (e.g. confidence intervals)
	x	For null hypothesis testing, the test statistic (e.g. *F*, *t*, *r*) with confidence intervals, effect sizes, degrees of freedom and *P* value noted<br>*Give P values as exact values whenever suitable.*
x		For Bayesian analysis, information on the choice of priors and Markov chain Monte Carlo settings
x		For hierarchical and complex designs, identification of the appropriate level for tests and full reporting of outcomes
x		Estimates of effect sizes (e.g. Cohen's *d*, Pearson's *r*), indicating how they were calculated

Our web collection on statistics for biologists contains articles on many of the points above.

Software and code

Policy information about availability of computer code

Data collection	The ATAC-seq data was generated in this work and sequenced with an Illumina NextSeq 2000 (100 cycles, Pair-end 50 bp). The ChIP-seq data for Hoxa13 and Gli3 was obtained from public databases (GSE81356 and GSE133710, respectively) and the vertebrate genomes conservation from the UCSC genome browser (mm10.60way.phyloP60way.bw file downloaded from http://hgdownload.cse.ucsc.edu/goldenpath/mm10/phyloP60way/). The optical projection tomography (OPT) and light sheet images were obtained with custom built OPT/SPIM scanners.
Data analysis	Data analysis: Statistical analysis: R version 4.2.1 (R studio Version 2023.03.0+386); R packages ggplot2, ggdignif, Deseq2, ChIPseeker, EdgeR, pheatmap.<br>Bioinformatic analysis: FastQC (v0.11.9), cutadapt (v1.16.5), Bowtie2 (v2.4.2), BamTools Filter (v2.4.1), Picard MarkDuplicates (v2.18.2.2), Bedtools (v2.30.0), MACS2 (v2.1.1), Deeptools, RGT HINT-ATAC.<br>Image analysis: ImageJ, Nrecon (SkyScan), Amira software (Thermo Fisher Scientific), Biomedisa.

For manuscripts utilizing custom algorithms or software that are central to the research but not yet described in published literature, software must be made available to editors and reviewers. We strongly encourage code deposition in a community repository (e.g. GitHub). See the Nature Portfolio guidelines for submitting code & software for further information.

Data

Policy information about availability of data

All manuscripts must include a data availability statement. This statement should provide the following information, where applicable:

- Accession codes, unique identifiers, or web links for publicly available datasets
- A description of any restrictions on data availability
- For clinical datasets or third party data, please ensure that the statement adheres to our policy

The ATAC-seq data obtained in this work was deposited in GEO repository, accession number GSE231592 (https://www.ncbi.nlm.nih.gov/geo/query/acc.cgi?acc=GSM7291329)]. In addition, we also used the following published data: conservation scoring by phyloP (phylogenetic p-values) for 60 vertebrate genomes from the UCSC genome browser (http://hgdownload.cse.ucsc.edu/goldenpath/mm10/phyloP60way/) and ChIP-seq data from Hoxa13 (GSE81356 [https://www.ncbi.nlm.nih.gov/geo/query/acc.cgi?acc=GSE81356]) and Gli3 [GSE133710 (https://www.ncbi.nlm.nih.gov/geo/query/acc.cgi?acc=GSM3923170)]. The reference mouse genome used in this work was GRCm38/mm10..

Research involving human participants, their data, or biological material

Policy information about studies with human participants or human data. See also policy information about sex, gender (identity/presentation), and sexual orientation and race, ethnicity and racism.

Reporting on sex and gender	*Use the terms sex (biological attribute) and gender (shaped by social and cultural circumstances) carefully in order to avoid confusing both terms. Indicate if findings apply to only one sex or gender; describe whether sex and gender were considered in study design; whether sex and/or gender was determined based on self-reporting or assigned and methods used.*<br>*Provide in the source data disaggregated sex and gender data, where this information has been collected, and if consent has been obtained for sharing of individual-level data; provide overall numbers in this Reporting Summary. Please state if this information has not been collected.*<br>*Report sex- and gender-based analyses where performed, justify reasons for lack of sex- and gender-based analysis.*
Reporting on race, ethnicity, or other socially relevant groupings	*Please specify the socially constructed or socially relevant categorization variable(s) used in your manuscript and explain why they were used. Please note that such variables should not be used as proxies for other socially constructed/relevant variables (for example, race or ethnicity should not be used as a proxy for socioeconomic status).*<br>*Provide clear definitions of the relevant terms used, how they were provided (by the participants/respondents, the researchers, or third parties), and the method(s) used to classify people into the different categories (e.g. self-report, census or administrative data, social media data, etc.)*<br>*Please provide details about how you controlled for confounding variables in your analyses.*
Population characteristics	*Describe the covariate-relevant population characteristics of the human research participants (e.g. age, genotypic information, past and current diagnosis and treatment categories). If you filled out the behavioural & social sciences study design questions and have nothing to add here, write "See above."*
Recruitment	*Describe how participants were recruited. Outline any potential self-selection bias or other biases that may be present and how these are likely to impact results.*
Ethics oversight	*Identify the organization(s) that approved the study protocol.*

Note that full information on the approval of the study protocol must also be provided in the manuscript.

Field-specific reporting

Please select the one below that is the best fit for your research. If you are not sure, read the appropriate sections before making your selection.

[x] Life sciences [ ] Behavioural & social sciences [ ] Ecological, evolutionary & environmental sciences

For a reference copy of the document with all sections, see nature.com/documents/nr-reporting-summary-flat.pdf

Life sciences study design

All studies must disclose on these points even when the disclosure is negative.

Sample size	ISH was performed on 3 independent biological replicates per probe and developmental stage which was sufficient to verify consistency in patterns of gene expression. We used higher number of transgenic embryos, because depending on the transgene frequency of reporter activation may vary. We aimed to obtain at least 2 (for most of reporters 5-6) transgenic embryos with consistent reporter expression to identify a reproducible pattern of the reporter.<br>For the ATAC-seq analyses, we used two independent biological replicates per tissue, which is a consensus standard for high-throughput sequencing experiments. Each contained pooled tissue from two embryos to reduce variability.<br>For quantitative RT-PCR analysis, data variability was low and sample sizes that we used (n=2 for controls, n=4-5 for mutants) were sufficient to evaluate significant differences.
Data exclusions	No data exclusion

Replication	All attempts of replication in ISH and transgenic reports assay were successful. All ISH experiments were reproduced in at least two independent experiments including embryos from independent litters.
Randomization	No randomization involved in the study
Blinding	No blinding involved in the study

Reporting for specific materials, systems and methods

We require information from authors about some types of materials, experimental systems and methods used in many studies. Here, indicate whether each material, system or method listed is relevant to your study. If you are not sure if a list item applies to your research, read the appropriate section before selecting a response.

Materials & experimental systems

n/a	Involved in the study
☐	☒ Antibodies
☐	☒ Eukaryotic cell lines
☒	☐ Palaeontology and archaeology
☐	☒ Animals and other organisms
☒	☐ Clinical data
☒	☐ Dual use research of concern
☒	☐ Plants

Methods

n/a	Involved in the study
☒	☐ ChIP-seq
☒	☐ Flow cytometry
☒	☐ MRI-based neuroimaging

Antibodies

Antibodies used	anti-Tgfbr1 (Sigma-Aldrich #HPA056473), anti-actin (Abcam #ab179467) and HRP conjugated-anti-rabbit IgG (GE Healthcare # NA9340)
Validation	They are commercial antibodies

Eukaryotic cell lines

Policy information about cell lines and Sex and Gender in Research

Cell line source(s)	HEK 293T cells (ATCC #CRL-3216)
Authentication	They were taken from ATCC and no further authentication was done
Mycoplasma contamination	Not tested for mycoplasma
Commonly misidentified lines (See ICLAC register)	N/A

Animals and other research organisms

Policy information about studies involving animals; ARRIVE guidelines recommended for reporting animal research, and Sex and Gender in Research

Laboratory animals	Mus musculus, with mixed C57BL/6-FBV-J mixed background. The lines carrying Tgfbr1 a null allele, the Tgfbr1 floxed allele and the Cdx2-CreERT lines had been described before and the references specified in the manuscript. The Tgfbr13ex3 allele was generated in this study together with a description of its characterization. The mice used in this work were maintained on a 12 hour light/dark cycle, at 22ºC with humidity ranging between 40 and 60%. Mice used for matings were between 3 and 6 months old.
Wild animals	N/A
Reporting on sex	At the stage the embryos were analyzed there are no major differences between sexes and therefore the sex of the specific embryos used in our experiments was not determined.
Field-collected samples	N/A
Ethics oversight	All animal procedures were performed in accordance with Portuguese (Portaria 1005/92) and European (directive 2010/63/EU) legislations and guidance on animal use in bioscience research. The project was reviewed and approved by the Ethics Committee of “Instituto Gulbenkian de Ciência” and by the Portuguese National Entity “Direcção Geral de Alimentação Veterinária” (license reference: 014308).

Note that full information on the approval of the study protocol must also be provided in the manuscript.

Plants

Seed stocks	*Report on the source of all seed stocks or other plant material used. If applicable, state the seed stock centre and catalogue number. If plant specimens were collected from the field, describe the collection location, date and sampling procedures.*
Novel plant genotypes	*Describe the methods by which all novel plant genotypes were produced. This includes those generated by transgenic approaches, gene editing, chemical/radiation-based mutagenesis and hybridization. For transgenic lines, describe the transformation method, the number of independent lines analyzed and the generation upon which experiments were performed. For gene-edited lines, describe the editor used, the endogenous sequence targeted for editing, the targeting guide RNA sequence (if applicable) and how the editor was applied.*
Authentication	*Describe any authentication procedures for each seed stock used or novel genotype generated. Describe any experiments used to assess the effect of a mutation and, where applicable, how potential secondary effects (e.g. second site T-DNA insertions, mosiacism, off-target gene editing) were examined.*
